# Identifying binge drinkers based on parenting dimensions and alcohol-specific parenting practices: building classifiers on adolescent-parent paired data

**DOI:** 10.1186/s12889-015-2092-8

**Published:** 2015-08-05

**Authors:** Rik Crutzen, Philippe J. Giabbanelli, Astrid Jander, Liesbeth Mercken, Hein de Vries

**Affiliations:** Department of Health Promotion, CAPHRI School for Public Health and Primary Care, Maastricht University, P.O. Box 616, 6200 MD Maastricht, The Netherlands; UKCRC Centre for Diet and Activity Research, MRC Epidemiology Unit, University of Cambridge School of Clinical Medicine, Institute of Metabolic Science, CB2 0QQ Cambridge, UK; Interdisciplinary Research in the Mathematical and Computational Sciences (IRMACS) Centre, Simon Fraser University, Burnaby, Canada

**Keywords:** Classifiers, Parenting practices, Parenting dimensions, Binge drinking

## Abstract

**Background:**

Most Dutch adolescents aged 16 to 18 engage in binge drinking. Previous studies have investigated how parenting dimensions and alcohol-specific parenting practices are related to adolescent alcohol consumption. Mixed results have been obtained on both dimensions and practices, highlighting the complexity of untangling alcohol-related factors. The aim of this study was to investigate (1) whether parents’ reports of parenting dimensions and alcohol-specific parenting practices, adolescents’ perceptions of these dimensions and practices, or a combination are most informative to identify binge drinkers, and (2) which of these parenting dimensions and alcohol-specific parenting practices are most informative to identify binge drinkers.

**Methods:**

Survey data of 499 adolescent-parent dyads were collected. The computational technique of data mining was used to allow for a data driven exploration of nonlinear relationships. Specifically, a binary classification task, using an alternating decision tree, was conducted and measures regarding the performance of the classifiers are reported after a 10-fold cross-validation.

**Results:**

Depending on the parenting dimension or practice, parents’ reports correctly identified the drinking behaviour of 55.8 % (using psychological control) up to 70.2 % (using rules) of adolescents. Adolescents’ perceptions were best at identifying binge drinkers whereas parents’ perceptions were best at identifying non-binge drinkers.

**Conclusions:**

Of the parenting dimensions and practices, rules are particularly informative in understanding drinking behaviour. Adolescents’ perceptions and parents’ reports are complementary as they can help identifying binge drinkers and non-binge drinkers respectively, indicating that surveying specific aspects of adolescent-parent dynamics can improve our understanding of complex addictive behaviours.

## Background

Binge drinking among adolescents (i.e., having 4/5 or more standard drinks of alcohol in one occasion for a girl/boy) is associated with poor school performance and involvement in other health risk behaviours such as riding with a driver who had been drinking, being a victim of dating violence, and using illicit drugs [1]. In the Netherlands, 57 % of the 16 year old and 62 % of the 17-18 year old engaged in binge drinking within 30 days [2]. This was legal, because Dutch adolescents were allowed to buy low-strength alcoholic beverages (e.g., wine, beer) at the age of 16 (which has changed to 18 in the new law by January 1^st^ 2014). This left Dutch parents in the situation that their children were allowed to buy alcoholic beverages, while parents were still responsible for their child’s health and behaviour (as they were only considered to be an adult as of the age of 18). In such a situation, both parenting in general as well as alcohol-specific parenting practices become highly important.

First, parenting can be viewed more generally as a constellation of attitudes and beliefs that create an emotional climate and determine the interactions between parent and child [3]. The current study includes three established dimensions of parenting, using the labels of Barber et al. [4]: support, psychological control, and one aspect of behavioural control assessing parental knowledge of their children’s activities. Parental support concerns parents’ affectionate qualities (e.g., being responsive to their child), whereas psychological control refers to parents being intrusive and manipulative regarding their children’s thoughts and feelings [4]. Van Zundert et al. [5] found that support and behavioural control were not related to adolescent alcohol use, whereas Barnes et al. [6] found no direct effect of support, but a protective effect of behavioural control regarding alcohol misuse. Also Donath et al. [7] found that support had no predictive power regarding binge drinking. However, another longitudinal study revealed that if adolescents perceived their parents to be supportive and controlling their behaviour, then they were less likely to have tried drinking or to be heavy drinking three years later [8]. Psychological control did not increase the likelihood that adolescents drink heavily [9]. A study based on parents’ reports found a protective effect of support for girls, but not for boys [10]. Hence, previous studies based on adolescents’ perceptions of parenting dimensions revealed mixed results, and insights based on parents’ reports are limited.

Second, there are alcohol-specific parenting practices, such as setting rules and communicating with children about alcohol. These are goal-directed behaviours used by parents to influence their children’s behaviours (e.g., binge drinking). In adolescents, perceiving that parents did not approve of drinking was associated with lower levels of heavy episodic drinking [11]. In another study, alcohol-specific rules, based on adolescents’ perceptions and reports of both parents, were very strongly negatively related to adolescents’ drinking [12]. However, parents reported that they imposed stricter rules in comparison with adolescents’ perceptions [13]. Alcohol-specific communication (e.g., talking with the child about how to resist peer pressure), as reported by parents, had trivial effects on drinking initiation [14], and excessive alcohol use and related problems of adolescents [15]. However, adolescents’ perceptions of alcohol-specific communication predicted subsequent adolescent’s alcohol use [16] and was associated with binge drinking and alcohol-related problems [17]. In other words, findings regarding alcohol-specific parenting practices are not univocal and depend on the person reporting them (e.g., adolescents or parents). This is indicative of the complexity involved in analysing alcohol-related factors.

In this study, we use the computational technique of *data mining* to explain the complex mechanisms underlying binge drinking [18]. This differs from the statistical approaches such as regressions that are traditionally used in analyses of alcohol-related factors, and (addictive) behaviours in general [19]. In data mining, the computer learns the relationships between factors by being provided many cases (e.g., data from *all* participants, independent of whether they are binge drinkers). For example, a certain number of participants completes a survey (e.g., about parenting dimensions) and reports whether they are binge drinkers: the computer would then learn from that data how the answers on the survey connect to the drinking status, without having to assume that the connection takes a specific mathematical shape (e.g., a linear function). This advantage was highlighted by Dierker and colleagues in their comparison of techniques used in research on substance abuse. They noted that computational techniques “allow for a data driven exploration of nonlinear relationships […] and have the potential to fit numerous interactions that cannot be handled as efficiently with either traditional regression techniques or other pattern centered methods” [20].

**Fig. 1:**
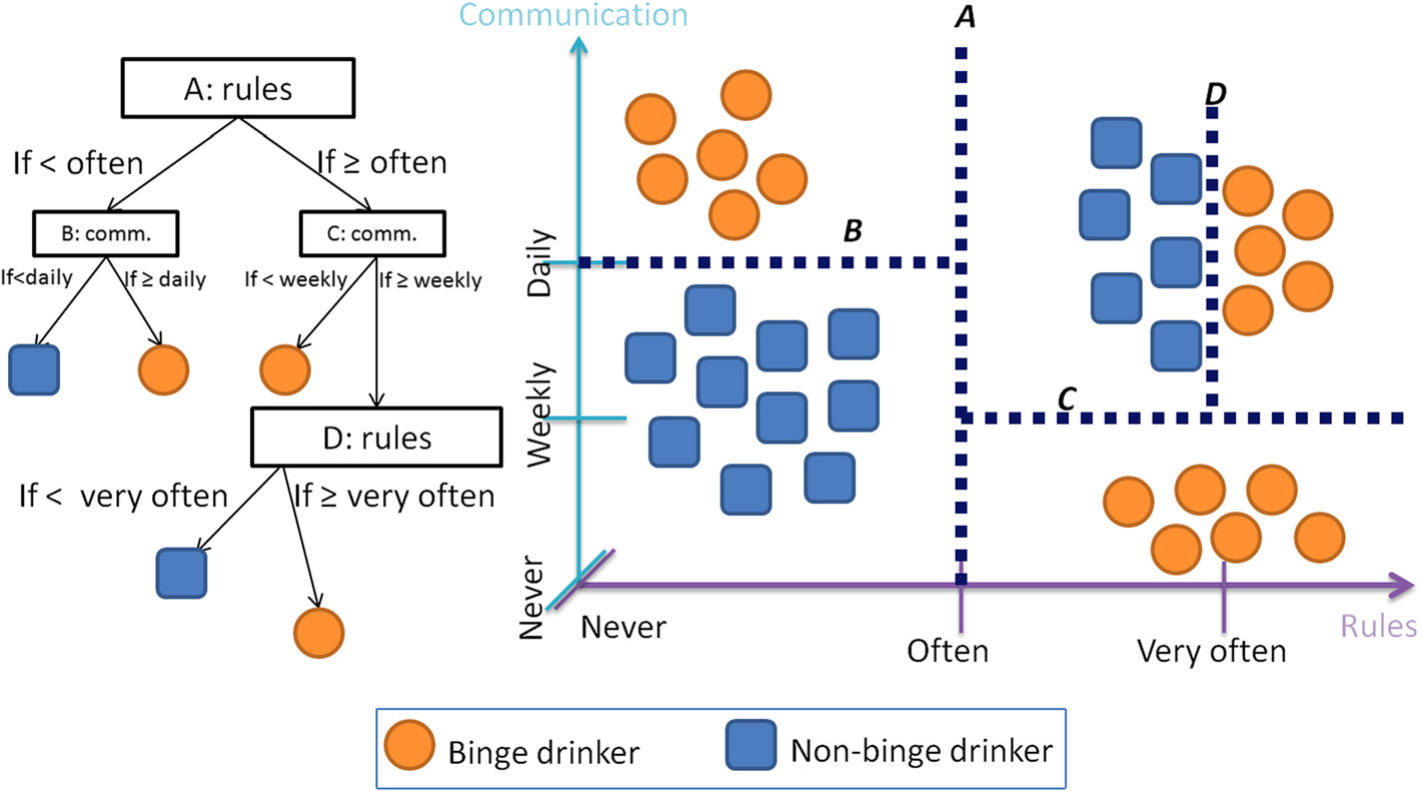
The training set is composed of individuals with a known target behaviour (binge drinker or not) and with known variables (e.g., rules and communication). In one approach to build a classifier (on the left), the computer automatically and repeatedly devides the training (on the right). This specific approach produces a decision tree, where a path from the root to a target behaviour corresponds to successive cuts in the training set

Several tools exist within data mining, depending on what has to be learned. In this study, we use *classifiers*. Intuitively, a classifier is a function that assigns labels to individuals (e.g., binge drinker or not) based on certain features (e.g., alcohol-specific parenting practices). Conceptually, the computer is first provided with individual-level cases (known as *training set*) in which individuals have known target behaviour (e.g., it is known whether someone is a binge drinker or not) and variables (e.g., alcohol-specific parenting practices), in order to learn how the variables are connected to the target behaviour. This results in a *classifier*, which can then be used to infer the unknown target behaviour of a new case. One possible approach to the creation of a classifier is exemplified in Fig. 1. The classifiers developed here are used to identify binge drinkers based on parents’ reports of parenting dimensions and alcohol-specific parenting practices, and adolescents’ perceptions.

Few studies have previously used classifiers to investigate drinking. These studies have focused on the identification of risk factors, for example regarding harmful alcohol use in Royal Australian Navy veterans [21], college drinking [18], or alcohol-withdrawal seizures [22]. This study is the first to use advanced data mining algorithms to examine how drinking in adolescents is influenced by parenting dimensions and alcohol-specific parenting practices. This is achieved by investigating (1) whether parents’ reports of parenting dimensions and alcohol-specific parenting practices, adolescents’ perceptions of these dimensions and practices, or a combination are most informative to identify binge drinkers, and (2) which of these parenting dimensions and alcohol-specific parenting practices are most informative to identify binge drinkers. Given the differences between mothers and fathers in terms of parenting, both in terms of quantity and nature [23], this study also explores whether adolescents’ perceptions of the mother or father are more useful in identifying binge drinkers.

## Methods

### Participants and procedure

Parents were recruited through an online panel (i.e., http://www.anniksystems.com/our-solutions-3/research-services/). Annik Systems works globally with over 240 panels to assist research with hard-to-reach audiences. Members of these panels previously expressed willingness to participate in business-, market-, and scientific research studies and provided their socio-demographic information. For this study, participants of Dutch panels with children between the age of 16 to 18 years were invited to participate in our study in September 2012. Self-reports in the survey were used to double-check whether parents had a child between 16 and 18 years old. Dyads of an adolescent and one of their parents were required for the study at hand. Therefore, parents were made aware that adolescent participation was required for this study. A total of 784 parents volunteered and, for 526 of them (67.1 %), their child participated. After data cleaning (i.e., discharging incomplete entries, checking for unreliable answers on variables and the time it took participants to complete the questionnaire), data for 499 adolescent-parent dyads (94.9 %) were used for the current study (Table 1).

**Table 1:** Socio-demographic characteristics of adolescent-parent dyads (N = 499)

Adolescents	Number	Percent
Male	249	49.9
High educational background	257	51.5
Dutch nationality	486	97.4
Age	M = 16.8	SD = 0.82
Parents		
Male	180	36.1
High educational background	165	33.1
Dutch nationality	474	95.0
Age	M = 47.4	SD = 5.75

Parents had to complete the measures described below (except for binge drinking) and, subsequently, their child was invited to participate (either right away or at a more convenient moment) and complete the same measures. The wording of the items was adjusted based on the person that completed the measures (e.g., “your child”, “your father”, “your mother”). Adolescents were asked separately for perceptions of their mother and perceptions of their father.

### Ethics approval

Ethics approval of the Regional Medical Ethics committee in the Netherlands was not necessary, because participants in this study were not subjected to procedures or required to follow certain rules of behaviour [24]. Informed consent was obtained before participation in the online survey. The panel complied with the Code of Standards and Ethics for Market, Opinion, and Social Research [25] as well as the ICC/ESOMAR Code on Market and Social Research [26].

### Measures

#### Binge drinking

Binge drinking was assessed with an open-ended question asking adolescents how many binge drinking occasions they had in the past 30 days. It was explained to them that binge drinking means having 4/5 or more standard drinks of alcohol in one occasion for a girl or boy respectively [27]. A standard drink in most on premise locations in the Netherlands contains 10 g of alcohol [28]. Adolescents were identified as binge drinkers if they reported at least one binge-drinking occasion.

#### Parenting dimensions

Parenting dimensions were assessed using a validated Dutch questionnaire [29]. Such assessment of parenting dimensions can and is recommended to be completed by both parents and adolescents [30]. Nine, eight, and five items were used to respectively assess support (e.g., “I can count on my parents to help me out, if I have some kind of problem”), psychological control, (e.g., “When I get a poor grade at school, my parents make me feel guilty”) and behavioural control (e.g., “I need permission to leave the house during the evening”). Participants had to indicate on a five-point response scale to what extent they agreed with these items (1 = “totally disagree”; 5 = “totally agree”).

#### Alcohol-specific parenting practices

Alcohol-specific rules were assessed by means of a validated Dutch questionnaire [31], that has been used for both adolescents and parents. One item about rules concerning binge drinking was added to this questionnaire, resulting in a total of 11 items (e.g., “How often do you allow your child to come home tipsy?”). Participants could give an indication using a five-point response scale ranging from 1 (“never”) to 5 (“very often”). Alcohol-specific communication was assessed by means of a questionnaire consisting of 8 items [14]. These items covered different areas of communication about alcohol, such as talking with the child about how to resist peer pressure. Participants could give an indication using a five-point response scale ranging from 1 (“never”) to 5 (“daily”).

Table 2 presents the eigenvalue (to estimate the explained variance; the eigenvalues should be at least > 1 [32]) and McDonald’s omega with confidence intervals [95 % CI] (to estimate factor saturation; the value represents a less biased alternative to Cronbach’s alpha [33]) of all measures of parenting dimensions and alcohol-specific parenting practices. These indices foster comprehensive assessment of questionnaire quality [34].

**Table 2:** Eigen values and omega of alcohol-specific parenting practices and parenting dimensions

Measure	Eigenvalue	Omega (95 % CI)	Alpha
Parents' reports			
Rules	6.41	.93 (.92-.94)	.93
Communication	5.40	.93 (.91-.94)	.93
Support	5.44	.91 (.90-.93)	.91
Psychological control	3.73	.81 (.77-.84)	.81
Behavioural control	3.41	.85 (.82-.88)	.87
Adolescents' perceptions of mother			
Rules	5.47	.92 (.91-.93)	.92
Communication	5.62	.94 (.93-.95)	.94
Support	5.67	.92 (.91-.93)	.92
Psychological control	4.31	.87 (.84-.89)	.87
Behavioural control	3.59	.90 (.87-.91)	.90
Adolescents' perceptions of father			
Rules	5.92	.93 (.92-.95)	.93
Communication	5.85	.95 (.93-.96)	.95
Support	6.29	.95 (.94-.96)	.95
Psychological control	4.55	.89 (.87-.90)	.89
Behavioural control	3.91	.93 (.91-.94)	.93

### Analyses

In this study, we aimed at identifying the drinking status of individuals given different sets of variables reported either by the individual or by parents. Specifically, we performed one analysis to assess the extent to which the drinking status could be inferred using each of the five parenting dimensions and alcohol-specific parenting practices (rules, communication, support, psychological control, behavioural control), and for each of the five possible sources of reporting (adolescents’ perceptions and parents’ reports, parents’ reports only, adolescents’ perceptions of both parents/mother only/father only). This resulted in a total of 25 analyses. The gender of both the adolescent (49.9 % male, 50.1 % female) and the parent respondent (36.1 % male, 63.9 % female) were used in all of the 25 analyses. For example, when examining the extent to which binge drinking could be inferred from adolescents’ perceptions and parents’ reports of rules, then the gender of the adolescent and of the parent were also part of the dataset.

The behaviour of each individual was dichotomized as being a binge drinker or not; individuals who did not engage in any form of drinking or were only drinking moderately were categorized as non-binge drinkers. Consequently, this identification task is known as a *binary classification*, whereby we want to know to which one of the two groups each individual belongs. The few studies that performed a binary classification on alcohol-related factors have employed a variety of classifiers [18, 19, 21, 22, 35]. This study uses decision trees, as it is the most prevalent data mining technique in research on drinking behaviour and has also been applied to other cases of substance use such as smoking [20]. Specifically, we use an alternating decision tree, which improves the performance of decision trees using the boosting procedure [36]. Intuitively, the key difference between alternating decision trees and simpler ones (such as illustrated in Fig. 1) is that one new case would result in only one path from the root to an inferred behaviour in simpler trees, while that same case may result in multiple paths in an alternating decision tree.

Several metrics are used to evaluate binary classifiers. These metrics are derived from a *confusion matrix*, which is a comparison of the number of individuals deemed to be binge drinkers or not versus the real data. For example, the classifier could state that 90 individuals are binge drinkers and 10 are not, whereas the data says that 80 individuals are binge drinkers and 20 are not. From this, we derive all other measures: the accuracy (i.e., percentage of correctly classified instances), sensitivity (i.e., rate of correctly classified binge drinkers), and specificity (i.e., rate of correctly classified non binge drinkers). Our measures are reported using a standard procedure known as 10-fold cross-validation. In this procedure, “the data set is split into 10 parts of approximately equal sizes, and each part is used in turn for testing of a classifier built on the pooled remaining 9 parts” [37]. The main advantage of this procedure is that the performance of a classifier is evaluated on different instances than those used to build it, in which case it could perform artificially high.

We have recently emphasized the importance of full disclosure to maximize scrutiny, foster accurate replication, and facilitate future data syntheses (e.g., meta-analyses) [38, 39]. Therefore, non-identifiable data and the output of the analysis (including the confusion matrices) are available at https://osf.io/tbqy7/.

## Results

In Table 3, we report on the accuracy, where higher accuracy indicates better performance of the classifier. Most of the sample is made of non-binge drinkers (59.8 %) so identifying them correctly while not correctly identifying the binge drinkers (40.2 %) could still lead to a high accuracy. In this situation, we previously emphasized that we should seek a balance whereby we are able to identify a large number of individuals for both behaviours [19]. Consequently, Table 4 provides the performances for both binge drinkers (first row) and non-binge drinkers (second row), respectively known as sensitivity and specificity. The latter were consistently higher than the former.

**Table 3:** Accuracy of classifiers

Correctly identified cases (%) based on…	Rules	Communication	Support	Psychological control	Behavioural control
…adolescents' perceptions *and* parents' reports	66.4	63.6	61.4	58.0	55.6
…parents’ reports	70.2	62.6	70.2	55.8	59.8
…adolescents' perceptions of both parents	60.6	59.8	59.2	55.8	57.0
…adolescents' perceptions of mother only	63.6	64.8	61.4	56.4	55.6
…adolescents' perceptions of father only	62.4	60.2	58.2	53.4	59.8

**Table 4:** Sensitivity and specificity of classifiers^a^

Correctly identified binge drinkers/non-binge drinkers (%) based on	Rules	Communication	Support	Psychological control	Behavioural control
…adolescents' perceptions *and* parents' reports	53.7	44.3	30.8	18.9	15.9
	74.9	76.6	81.9	84.3	82.3
…parents' reports	58.2	33.8	7.5	31.8	17.4
	78.3	81.9	93.0	71.9	88.3
…adolescents' perceptions of both parents	64.7	33.8	21.4	20.9	20.4
	65.9	77.3	84.6	79.3	81.6
…adolescents' perceptions of mother only	55.7	31.3	20.4	18.9	36.8
	68.9	87.3	89.0	81.6	68.2
…adolescents' perceptions of father only	60.2	28.9	18.9	20.4	22.9
	63.9	81.3	84.6	75.6	84.6

^a^Performances for binge drinkers (first row; sensitivity) and non-binge drinkers (second row; specificity)

Of all parenting dimensions and alcohol-specific parenting practices, only rules can identify most adolescents correctly (i.e., both binge drinkers and non-binge drinkers). None of the other variables can identify most adolescents: they perform poorly in terms of identifying binge drinkers (i.e., sensitivity < 50 %; Table 4). The high overall accuracy is mostly due to the high accuracy regarding non-binge drinkers, which are more prevalent in the data.

The adolescents’ perceptions of the mother are better at identifying adolescents in general (Table 3) and non-binge drinkers (Table 4; second row) than perceptions of the father, except for behavioural control. Adolescents’ perceptions of the father with regard to rules and psychological control are better at identifying binge drinkers than perceptions of the mother (Table 4).

## Discussion

The aim of this study was to investigate (1) whether parents’ reports of parenting dimensions and alcohol-specific parenting practices, adolescents’ perceptions of these dimensions and practices, or a combination are most informative to identify binge drinkers, and (2) which of these parenting dimensions and alcohol-specific parenting practices are most informative to identify binge drinkers.

Using the technique of classifiers, this study revealed that parents’ reports of parenting dimensions and alcohol-specific parenting practices *can* be helpful to identify non-binge drinkers. Yet, adolescents’ perceptions of these dimensions and practices are most accurate when identifying binge drinkers. This demonstrates the complexity of parent-adolescent dynamics, as all family members experience alcohol-specific socialisation (e.g., rule setting, talking about alcohol use) [31]. The current study indicates that this is not only limited to children’s perceptions of parental drinking [40], but also relevant in the context of parenting dimensions and alcohol-specific parenting practices.

Adolescents’ perceptions of their mother’s parenting dimensions and alcohol-specific parenting practices were more useful to identify adolescents in general in comparison with perceptions of their father (Table 3). Gender-specific findings regarding hazardous drinking among offspring have been described previously in the context of parental alcohol misuse [41]. For example, boys with misusing mothers reported less alcohol consumption than other boys, while this was the opposite for those with misusing fathers.

Of the parenting dimensions and practices, rules were most informative to identify binge drinkers, despite possible differences in strictness between parents’ reports and adolescents’ perceptions [13]. This is in line with findings from a Delphi study among international experts, in which advising parents to have clear and consistent rules was considered to be an “extremely important” strategy to reduce binge drinking among this target group [42]. Rules are also an important theme within the Australian parenting guidelines for adolescent alcohol use [43]. It is recommended, therefore, that future interventions focus on how parents should set appropriate rules concerning alcohol use. For example, by adding a parental component to interventions that are primarily targeted at adolescents themselves [44]. The high specificity regarding the parenting dimensions is in line with the idea of authoritative parenting being a protective factor for mental health in adolescence [45]. Although the focus of the current study is on parenting dimensions and practices, we do acknowledge that that parental alcohol and tobacco use can influence adolescent behaviour directly. Moreover, parental drinking is also related to less engagement in alcohol-specific parenting practices [12, 14]. In other words, the influence of parental behaviour can be direct as well as indirect (e.g., mediated via parenting practices).

The accuracy of classifiers to identify the drinking status based on rules can be deemed relatively high given the complexity of drinking behaviour. Nevertheless, there are numerous obstacles to comparing accuracy between studies. While numerous studies have examined how drinking behaviour is influenced by dimensions and practices such as rules, this is rarely seen as a binary classification task. There are only a handful of such studies [18, 21, 22, 35], which makes comparisons challenging. Moreover, these few studies do not report performances in the same way. Studies in the behavioural sciences may report specificity and sensitivity, while studies in the medical sciences may report the Receiver Operating Characteristics (i.e., plotting sensitivity and specificity against each other as a function of some threshold criterion) and the Area Under the Curve (AUC) [46]. Even when considering the few studies that (i) perform a binary classification task on drinking and (ii) report the AUC [18, 21, 22], it may not be possible to adequately compare the results. Indeed, the AUC values false positives and negatives depending on the classifier under use [47]. Given that a variety of classifiers has been used, it is thus not possible to properly compare their performances. In contrast to past studies that reported performances using a few measures, our study of binge drinking is the first to fully disclose the performance of its classifiers. By generalizing such practice, it would become possible to compare the accuracy across studies, which should contribute to better assessing which dimensions and practices have the most impact on drinking behaviour.

A limitation of this study is that causal relationships between parenting dimensions and alcohol-specific parenting practices, and binge drinking cannot be unravelled due to the use of cross-sectional data from a potentially selective sample. However, this study was aimed at *identification* by means of classifiers, which concerns explaining instead of predicting behaviour. Moreover, our sample consisted mainly of participants with the Dutch nationality. This may not be representative for the Dutch population as 21.1 % of the Dutch population in 2013 consisted of immigrants (according to Statistics Netherlands: http://statline.cbs.nl/Statweb/?LA=en). Of all immigrants, more than half are from non-western countries, mainly from Turkey and Morocco. Immigrants from these countries are mainly Muslim, which might have an impact on parenting dimensions and alcohol-specific parenting practices. However, we only have information about nationality, so we could not verify whether people with Dutch nationality are also of Dutch ethnicity.

Another point worth rising is that this study was conducted before the change in law (i.e., increasing the legal alcohol buying age from 16 to 18). We still consider the findings as being relevant, because although parents often think their influence is decreasing when adolescents enter the legal alcohol buying age, a previous study suggests that their influence persists, even in situations where they are not present [Jander A, Mercken L, Crutzen R, Candel M, De Vries H: Parents’ influence on alcohol use among 16 to 18 year old Dutch adolescents: impact of alcohol specific rules and communication, submitted]. Moreover, adolescents from other countries are allowed to buy alcohol at the age of 16 (e.g., Austria, Denmark, Germany) [7]. Investigating differences between countries in terms of identifying binge drinkers based on parenting dimensions and alcohol-specific parenting practices might be an interesting avenue for further research. Not only because of differences in legal alcohol buying age, but also because countries differ in terms of drinking cultures [48].

## Conclusion

Of the parenting dimensions and practices, rules are particularly informative in understanding drinking behaviour. Adolescents’ perceptions and parents’ reports are complementary as they can help identifying binge drinkers and non-binge drinkers respectively, indicating that surveying specific aspects of adolescent-parent dynamics can improve our understanding of complex addictive behaviours.

## Abbreviations

AUC: Area Under the Curve
CI: Confidence Interval
